# Evaluation of cardiac masses by real-time perfusion imaging echocardiography

**DOI:** 10.1186/s12947-015-0018-3

**Published:** 2015-05-02

**Authors:** Eliza K Uenishi, Márcia A Caldas, Jeane M Tsutsui, Maria C D Abduch, João C N Sbano, Roberto Kalil Filho, Wilson Mathias

**Affiliations:** Heart Institute (InCor), University of Sao Paulo Medical School, São Paulo, Brazil; Heart Institute (InCor), University of Sao Paulo Medical School and Fleury Group, Av. Dr. Enéas de Carvalho Aguiar, 44, São Paulo, 05403-000 Brazil

**Keywords:** Cardiac tumor, Contrast echocardiography, Dipyridamole stress echocardiography

## Abstract

**Background:**

Diagnosis of cardiac masses is still challenging by echocardiography and distinguishing tumors from thrombi has important therapeutical implications. We sought to determine the diagnostic value of real-time perfusion echocardiography (RTPE) for cardiac masses characterization.

**Methods:**

We prospectively studied 86 patients, 23 with malignant tumors (MT), 26 with benign tumors (BT), 33 with thrombi and 6 with pseudotumors who underwent RTPE. Mass perfusion was analyzed qualitatively and blood flow volume (*A*), blood flow velocity (*β*), and microvascular blood flow (*A x β*) were determined by quantitative RTPE.

**Results:**

Logistic regression models showed that the probability of having a tumor increased by 15.8 times with a peripheral qualitative perfusion pattern, and 34.5 times with a central perfusion pattern, in comparison with the absence of perfusion. Using quantitative RTPE analysis, thrombi group had parameters of blood flow lower than tumor group. *A* values for thrombi, MT, and BT were 0.1 dB (0.01-0.22), 2.78 dB (1–7) and 2.58 dB (1.44-5), respectively; p < 0.05, while *A x β* values were 0.0 dB/s^−1^ (0.01–0.14), 2.00 dB/s^−1^ (1–6), and 1.18 dB/s^−1^ (0.52–3), respectively; p < 0.05. At peak dipyridamole stress, MT had greater microvascular blood volume than BT [*A* = 4.18 dB (2.14-7.93) versus *A* = 2.04 dB (1.09-3.55); p < 0.05], but no difference in blood flow [*Axβ* = 2.46 dB/s^−1^ (1.42–4.59) versus *Axβ* = 1.55 dB/s [1] (0.51-4.08); p = NS]. An *A* value >3.28 dB at peak dipyridamole stress predicted MT (AUC = 0.75) and conferred 5.8-times higher chance of being MT rather than BT.

**Conclusion:**

RTPE demonstrated that cardiac tumors have greater microvascular blood volume and regional blood flow when compared with thrombi. Dipyridamole stress was useful in differentiating MT from BT.

## Background

Cardiac masses are still a challenging issue during transthoracic echocardiography and can mainly be classified as tumors, thrombi or pseudotumors. Primary cardiac tumors are rarely found (0.001%) [1]. Histologically, 75% of tumors are benign, while 25% are malignant [2]. Secondary cardiac tumors or metastases are at least 100 times more frequent than primary tumors [3]. Intracardiac thrombi are blood clots that may also be erroneously detected as tumors and occur typically in areas of blood stasis, while pseudotumors are normal variations of cardiac structures, which can be confounded with other forms of cardiac masses. The etiology of a cardiac mass is crucial for therapeutic management, however they still pose a significant challenge in the clinical practice; also, an expeditious determination of cardiac masses nature has important practical implications in the clinical management of patients. The analysis of vascularity of these structures may be important for this purpose, and real-time perfusion echocardiography (RTPE) is an emerging approach that can be used to evaluate vascularity.

RTPE has been demonstrated to be a useful technique for the quantification of myocardial perfusion and determination of myocardial blood flow reserve [4-7]. Case reports and a single center study with a small sample of 16 patients have shown the possibility of evaluating vascularization of cardiac masses aiming to distinguish malignant from benign cardiac tumors, and also tumors from thrombi [8-14].

In this study, we sought to demonstrate the diagnostic value for characterization of cardiac masses through qualitative and quantitative analyses of RTPE.

## Methods

### Study patients

From July 2004 to October 2008, we prospectively studied 107 patients with cardiac masses detected by transthoracic echocardiography who underwent RTPE and subsequent diagnostic investigation of mass etiology. The study protocol was approved by the Ethical Committee of the University of São Paulo Medical School, and all patients provided written informed consent to participate. Exclusion criteria were pregnancy or breast feeding, intracardiac shunt, refusal or inability to sign the informed consent, severe obstructive pulmonary disease, second or third-degree atrioventricular block and history of allergy to components of the echocardiographic contrast agents. Patients in whom the diagnosis of cardiac mass could not be reached based on results of pathology, clinical data and other imaging technique (magnetic resonance imaging or computed tomography), were also excluded.

### Real-time perfusion echocardiography

After collecting the clinical history, a complete echocardiographic study was performed according to the recommendations of the American Society of Echocardiography [15]. Images were acquired with commercially available ultrasound systems (SONOS 5500 and IE33, Philips Medical Systems, Bothell, Washington, USA) equipped with S3 wide band transducer and contrast echo software with low mechanical index imaging.

Analysis of cardiac mass localization, size, quantity, echogenicity, mobility, relationships with adjacent structures and hemodynamic impairment was performed before contrast infusion. Imaging was performed in the apical 2-, 3- and 4-chamber views using power modulation mode with a mechanical index of 0.2, frame rate 25–30 Hz and continuous intravenous infusion of either lipid-encapsulated microbubbles Definity® (Lantheus Medical Imaging, Inc., N. Billerica, Massachusetts, USA) or PESDA (perfluorocarbon-exposed sonicated dextrose albumin). The formulation of PESDA has been described elsewhere [16]. Microbubble destruction was achieved using a packet of five high-intensity (mechanical index 1.5) pulses (flash). One vial of ultrasound contrast agent Definity® was diluted in 60 mL saline solution and infused continuously into the right antecubital vein after an initial bolus dose of 3 mL. PESDA was administered intravenously in a continuous fashion at a rate of 2–5 ml/min and prepared by diluting a suspension of 0.1 ml/kg of contrast in 80 ml of 5% dextrose.

Optimal gain and compression were obtained, and ultrasound beam focus was adjusted below the region of interest level. Image acquisition at baseline was obtained soon after optimization of settings and as soon as contrast opacification of the left ventricular cavity was considered homogeneous. A packet of five high intensity (mechanical index 1.5) flashes was manually deflagrated at a maximal visual contrast intensity to achieve microbubble destruction within the myocardium and cardiac masses. Contrast replenishment within the myocardial structures and cardiac masses was analyzed in low mechanical power (0.2) myocardial perfusion images including at least 15 cardiac cycles after the flash. Patients who agreed to undergo stress, were ≥18 years old and had no contraindication to dipyridamole underwent dipyridamole stress after acquisition of baseline images. A total dose of 0.84 mg/Kg of dipyridamole was infused during 10 minutes (peak). At the end of dipyridamole infusion, a second acquisition of images was performed using the same settings [17].

Continuous cardiac monitoring with one electrocardiographic lead, blood pressure, heart rate and 12-lead electrocardiography were registered at the 4th and 10th minutes after dipyridamole infusion, and after the test was terminated. After acquisition of peak images patients received aminophylline and were observed at rest for 30 minutes, until they returned to baseline conditions.

### Qualitative analysis of mass perfusion

**Figure 1:**
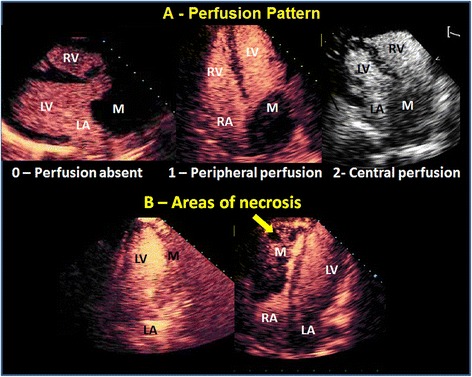
Qualitative analysis of mass perfusion demonstrating examples of perfusion pattern **(A)** and areas of necrosis **(B)**. LA = left atrium; RA = right atrium; LV = left ventricle; RV = right ventricle; M = cardiac mass.
- Areas of necrosis (areas with no perfusion in one specific region of the mass with perfusion around it): 0 – absent; 1 – present (Figure 1B).

Each set of digital images was reviewed by a sole experienced and independent observer (EKU), blinded to other study data. Starting from the first frame after the ultrasound destruction of microbubbles, cardiac masses were qualitatively analyzed during optimal cavity contrast enhancement based on a visual assessment of mass contrast enhancement. This assessment included the presence of perfusion, the velocity of perfusion replenishment after flash impulse, and the pattern of perfusion in the mass. A scoring system was established by considering the following parameters:

- Mass perfusion: 0 – perfusion absent; 1 – mild perfusion; 2 – moderate perfusion; 3 – intense perfusion;
- Replenishment perfusion velocity (post flash): 0 – perfusion absent; 1 – slow replenishment (some perfusion in the mass visualized 5 cardiac beats after flash); 2 – fast perfusion (some perfusion in the mass visualized before 5 cardiac beats after flash);
- Perfusion pattern: 0 – perfusion absent; 1 – peripheral perfusion (perfusion predominantly in the periphery of the mass, defined as its half external part); 2 – central perfusion (perfusion detected in the entire mass, including more than half internal part of the mass) (Figure 1A);

### Quantitative determination of mass perfusion

Off-line image analysis for perfusion was performed using commercially available software (Q-lab 5.0 Advanced Quantification Software – Philips Medical Systems, Bothell, WA, USA). Regions of interest were manually traced on the largest possible sample, in the planes where images of interest would be best visualized in end-systolic frames during baseline and, in those patients who underwent dipyridamole, at peak stress. Images frames were selected after 10 high-mechanical index frames (flashes), at the peak T-wave on the electrocardiogram, with careful exclusion of large vessels or areas of no perfusion. In addition, mass edges and pedicles were avoided.

**Figure 2:**
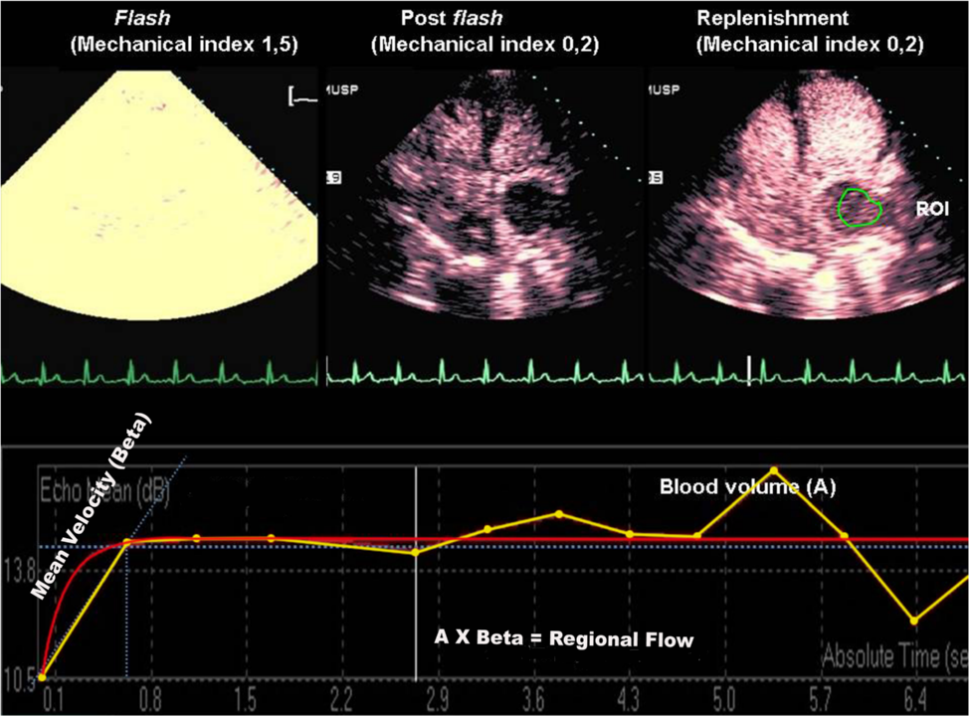
Images from real-time perfusion echocardiography (upper panels) in apical 4-chamber view in a patient with a malignant tumor in the left atrium. Image at left demonstrates the flash impulse high mechanical index followed by post-flash image (middle) and replenishment by contrast (image at right) with region of interest (ROI) manually traced in the tumor. Lower panel shows the time-intensity curve obtained by quantification software with estimation of myocardial blood volume at peak intensity (A), blood flow velocity (*β*) and regional blood flow (A x *β*). Yellow curve was defined by measurements of intensity at each time interval while red curve corresponded to exponential fitted time-acoustic intensity.

Acoustic intensity curves were built in an exponential function for analysis of 10 cardiac cycles on average for each sequence of images, starting from the flash, until the cardiac mass was completely replenished by contrast (Figure 2). Peak signal intensity plateau (A, dB) and signal intensity rate of rise (ß, s^−1^) – that is proportional to blood flow velocity - were calculated automatically by the software. To originate an index of mass microvascular blood flow, the product of A and ß was calculated [4]. For patients who underwent dipyridamole stress, mass blood flow reserve (Ax ß), and mass blood flow reserve (A) were calculated as the ratio of hyperemic to baseline parameter.

### Diagnostic confirmation of cardiac masses

Diagnostic determination of cardiac masses was performed by anatomical pathology in patients who underwent surgery or, in those who died, by autopsy [2]. The diagnosis of rhabdomyomas was confirmed by other cardiovascular imaging modality (magnetic resonance imaging or computed tomography), clinical association with tuberous sclerosis and serial echocardiographic demonstration of mass regression. The diagnosis of lipoma was performed by biopsy or magnetic resonance imaging with fat saturation approach. The diagnosis of cardiac thrombi was performed based on history and clinical presentation, including atrial flutter, atrial fibrillation, atrial dilatation, valvar stenoses and areas of left ventricular akinesia or diskinesia, associated with serial echocardiography findings of thrombus resolution after anticoagulation. Pseudotumor was defined as an anatomical variation or other cardiac abnormality (megaesophagus, endomyocardialfibrosis, abscess) not characterized as a cardiac tumor [18].

### Statistical analysis

All the variables were descriptively analyzed. Quantitative variables were analyzed by observing minimum and maximum values, and by calculating means, standard deviations and quartiles (percentile 25, median and percentile 75). Absolute and relative frequencies of qualitative variables were calculated. Means of groups were compared using unifactorial variance analyses, multiple comparisons were done with Bonferroni’s test. Kruskal-Wallis nonparametric test was used for variables with distribution not considered normal, and for multiple comparisons Dunn’s test was used.

Homogeneity between proportions was tested using chi-square or Fisher’s exact test (whenever expected frequencies were smaller than 5). Analysis of variance with repeat measures checked the behavior of groups along evaluations. For variables considered not normally distributed we used Wilcoxon’s nonparametric test. Cut-off values of variables were obtained using ROC (receiver-operator characteristic) curves. Intraobserver concordance was evaluated using Kappa’s coefficient.

Significance level was established at 5%.

## Results

**Figure 3:**
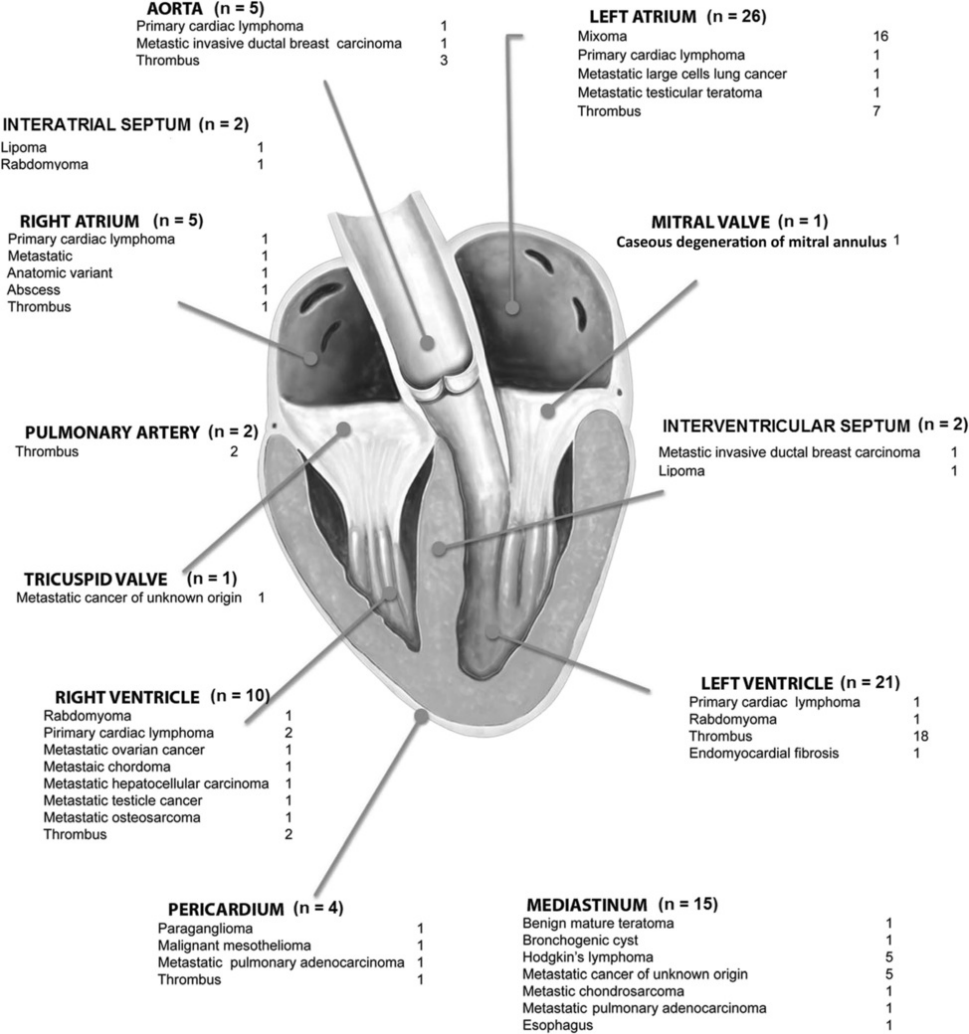
Distribution of type of cardiac masses and its distribution in the 86 studied patients.

Out of 107 initially enrolled patients, 21 were excluded because there was no definitive diagnostic confirmation of cardiac mass etiology. The final population consisted of 86 patients with mean age 49.3 ± 16.5 years, 49 (57%) female. Twenty-three (27%) patients had malignant (primary or secondary) tumors, 24 (28%) benign tumors, 33 (38%) thrombi, and 6 (7%) pseudotumors. The characteristics of patients and types of tumors are described in Tables 1 and 2. Localization and distribution of cardiac masses are seen in Figure 3. A total of 5 patients had more than one cardiac mass (2 patients with thrombi, 2 patients with rhabdomyoma and 1 patient with primary cardiac lymphoma). All the remaining patients had only 1 cardiac mass. Mean area size of malignant tumors was 16.1 cm^2^ (4.1 cm^2^ to 48.5 cm^2^), of benign tumors was 5.5 cm^2^ (1.9 cm^2^ to 40.1 cm^2^) and of thrombi was 6.4 cm^2^ (1.2 cm^2^ to 32.2 cm^2^).

**Table 1:** **Clinical characteristics of patients with malignant tumors, benign tumors, thrombi and pseudotumors**

**Variables**	**Malignant (n = 23)**	**Benign (n = 24)**	**Thrombi (n = 33)**	**Pseudotumors (n = 6)**	**P**
Age (years)	45.7 ± 19.2	46.8 ± 19.3	53.0 ± 16.2	52.5 ± 11.0	†
Female gender	12 (52.2%)	16 (64%)	17 (51.5%)	4 (57.1%)	†
Weight (Kg)	69.2 ± 20.6	63.0 ± 20.0	63.11 ± 11.5	77.7 ± 21.3	†
Height (cm)	165.6 ± 12.2	158.9 ± 13.7	164.0 ± 9.2	163.2 ± 10.1	†
Body mass index (Kg/m^2^)	24.8 ± 5.3	24.4 ± 6.2	23.4 ± 3.4	29.1 ± 7.1	†
Cardiovascular history					
Hypertension	7 (30.4%)	13 (52%)	15 (45.5%)	3 (42.8%)	†
Diabetes	4 (17.4%)	4 (16%)	4 (12.1%)	2 (28.6%)	†
Coronary artery disease	1 (4.3%)	3 (12%)	9 (27.3%)	1 (14.3%)	†
Dilated cardiomyopathy	0	1 (4%)	9 (27.3%)	2 (28.6%)	*
Congestive heart failure	3 (13.0%)	4 (16%)	17 (51.5%)	3 (42.8%)	*
Atrial fibrillation	1 (4.3%)	1 (4%)	4 (12.1%)	0	†
Embolic event	2 (8.7%)	0	4 (12.1%)	0	†
Cerebrovascular accident	1 (4.3%)	4 (16%)	3 (9.0%)	1 (14.3%)	†
Valvular heart disease	1 (4.3%)	1 (4%)	9 (27.3%)	0	*
Signs and symptoms					
Dyspnea	15 (65.2%)	5 (20%)	22 (66.7%)	3 (42.8%)	†§
Fever	1 (4.3%)	0	2 (6.0%)	1 (14.3%)	†
Peripheral edema	8 (34.8%)	0	10 (30.3%)	2 (28.6%)	*§
Dysphagia	4 (17.4%)	1 (4%)	0	0	*
Chest pain	7 (30.4%)	5 (20%)	3 (9.0%)	1 (14.3%)	†
Syncope	1 (4.3%)	1 (4%)	0	0	†
Odynophagia	3 (13.0)	0	0	0	*§
Weight loss	12 (52.2%)	0	7 (21.2%)	0	*§
Lipothymia	4 (17.4%)	2 (8%)	2 (6.0%)	0	†

† = not significant between groups; * = p < 0.001 between malignant tumors, benign tumors, thrombi and pseudotumors; § = p < 0.05 between malignant tumors and benign tumors.

**Table 2:** **Types of cardiac masses in the study population (also in Figure** **2**)

**Malignant cardiac tumors**	
**Primary**	
Primary cardiac lymphoma	3
Malignant pericardium mesothelioma	1
**Secondary (metastases)**	
Mediastinal – Lymphoma	5
Mediastinal neoplasia of uncertain origin	1
Pulmonary adenocarcinoma	3
Not-small cell pulmonary carcinoma	1
Ductal breast carcinoma	1
Malignant testicular teratoma	1
Ovarian cancer (unknown cellular type)	1
Hepatocellular carcinoma	1
Sacral chordoma	1
Chondrosarcoma	1
Osteosarcoma	1
Prostate adenocarcinoma	1
Metastasis of unknown origin	1
**Benign cardiac tumors**	
Myxoma	16
Rhabdomyoma	3
Paraganglioma	1
Lipoma	2
Bronchiogenic cyst	1
Mature teratoma (mediastinal)	1
**Thrombi**	
Intracardiac thrombi	33
**Pseudotumors**	
Abscess in right atrial lateral wall	1
Liver (diaphragm muscle paralysis)	1
Megaesophagus	1
Endomyocardiofibrosis	1
Caseous degeneration of mitral annulus	1
Anatomical variant (supra hepatic vein emerging into right atrium and left superior vena cava in coronary sinus)	1

### Qualitative analysis of mass by RTPE

In all patients cardiac mass was evaluated by qualitative RTPE. Distribution of cardiac masses according to qualitative perfusion scores is displayed in Table 3. Analysis of the relationship between qualitative parameters and groups (thrombi and tumor) shows that thrombi group had higher number of cases with zero perfusion score, in contrast with tumor group, in which the prevailing score was 1 (mild perfusion). Similarly, replenishment perfusion velocity in thrombi group was mostly equal to zero, while in tumor group the most frequent score was 1 (slow replenishment). The Kappa concordance coefficient for each variable shows that there was good correlation of scores with type of cardiac mass (tumor or thrombi) for mass perfusion (0.65; p < 0.001); replenishment perfusion velocity (0.643; p < 0.001) and areas of necrosis (0.468; p < 0.001). The correlation for perfusion pattern was suboptimal (0.379; p < 0,001). Logistic regression models showed that the probability of having a tumor increased by 15.8 times with a peripheral perfusion pattern, and 34.5 times with a central perfusion pattern, in comparison with the absence of perfusion. There was no association between perfusion pattern (either central or peripheral) and type of tumor. When areas of necrosis (absence of perfusion in one specific region of the mass with perfusion around it) were present, the probability of having a tumor was 6.7 times higher than when these were absent. Intraobserver variability was 20% considering perfusion score and perfusion velocity; 25% for areas of necrosis, and 45% for perfusion pattern. Intraobserver overall variability was 35%.

**Table 3:** **Qualitative real-time perfusion echocardiography scoring of cardiac masses (confirmed to be tumors or thrombi) as assessed by the presence, velocity, and pattern of perfusion**

**Scores**	**Groups**	**Presence of perfusion**	**Velocity**	**Perfusion pattern**	**Areas of necrosis**
0	Thrombi	27	27	27	31
	Tumors	11	9	9	37
1	Thrombi	6	0	4	2
	Tumors	33	10	21	16
2	Thrombi	0	6	2	--
	Tumors	6	34	23	--
3	Thrombi	0	--	--	--
	Tumors	3	--	--	--

Scores: **Perfusion**: 0 (absence); 1 (mild); 2 (moderate); 3 (intense). **Perfusion velocity**: 0 (absence); 1 (fast replenishment); 2 (slow replenishment)). **Perfusion pattern**: 0 (absence); 1 (peripheral); 2 (central). **Areas of necrosis**: 0 (absent); 1 (present).

### Quantitative analysis of mass by RTPE

Quantitative analysis was feasible in 73 (85%) patients. In 13 (15%) patients quantitative analysis was not possible due to technical difficulties, such as reduced mass size, excessive mass mobility (n = 5), and presence of acoustic shadowing over the mass (n = 8).

**Figure 4:**
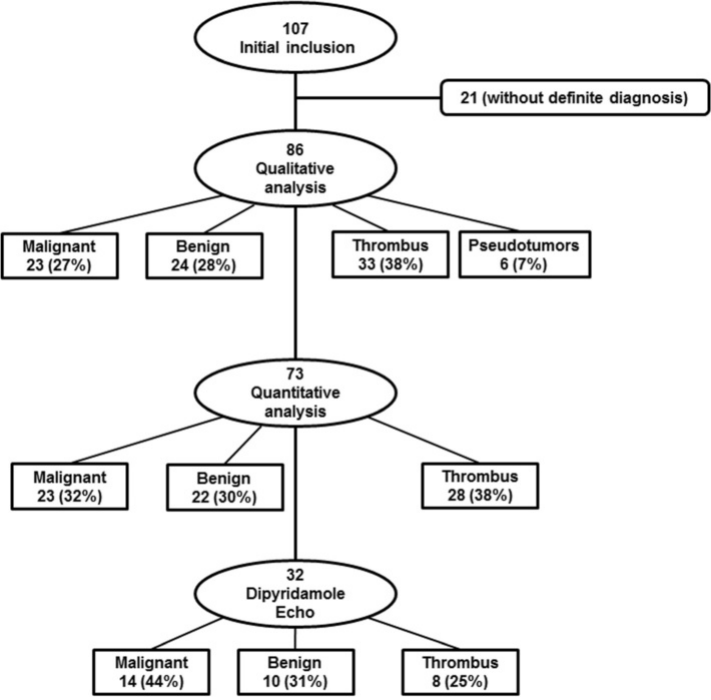
Flow chart showing patients’ selection for qualitative and quantitative real-time perfusion echocardiography.

Among the 73 patients with quantitative analysis of cardiac mass by RTPE, 23 (32%) had malignant tumors, 22 (30%) benign tumors, and 28 (38%) thrombi (Figure 4). The group thrombi showed significantly lower microvascular blood volume (A) and microvascular blood flow (A × *β*) values when compared with the groups with malignant and benign tumors. A and A × *β* did not show any significant differences between patients with malignant and benign tumors (Table 4).

**Table 4:** **Median and quartile values of quantitative perfusion variables in patients with malignant tumors, benign tumors and thrombi**

**Variable**	**Malignant tumors (n = 23)**			**Benign tumors (n = 22)**			**Thrombi (n = 28)**			
	**P25**	**MEDIAN**	**P75**	**P25**	**MEDIAN**	**P75**	**P25**	**MEDIAN**	**P75**	**p**
A (dB)	1.31	2.78	7.0	1.24	2.58	4.55	0.01	0.08	0.22	*†
Ax β (dB/s^−1^)	0.99	2.0	5.58	0.45	1.18	3.4	0.01	0.03	0.14	*†

A: Blood volume; A × β: Regional microvascular blood flow.

* = p < 0.001 between tumors and thrombi; † = not significant between malignant tumors and benign tumors.

**Figure 5:**
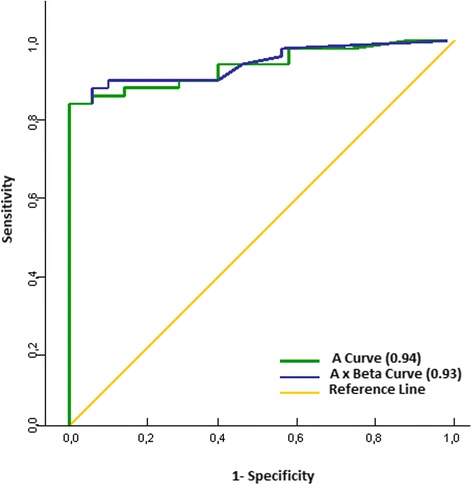
Receiver operator curves for A (blue line) and Ax*β* (green line) values for detection of tumors and thrombi groups.

A ROC curve was drawn to obtain the best cutoff value to help distinguish between thrombi and malignant/benign cardiac tumors. The parameter microvascular blood volume (A) showed an area under the curve (AUC) of 0.94. Thus, the A < 0.64 dB on RTPE predicts thrombus with a 93% sensitivity, 89% specificity, 81% positive predictive value, 95% negative predictive value, and 85% accuracy. The parameter microvascular blood flow (A × *β*) showed an AUC of 0.93 as predictor of thrombus, with 93% sensitivity, 84% specificity, 78% positive predictive value, 95% negative predictive value, and 87% accuracy (Figure 5). A cardiac mass with a value A × *β* < 0.30 dB/s^−1^ on RTPE had 68-times higher chance of being a thrombus rather than a malignant or benign tumor.

### Analysis of blood flow reserve by RTPE

Studies with dipyridamole for evaluation of tumor reserve was completed in 32 (44%) patients, 14 (44%) from the malignant tumor group, 10 (31%) from the benign tumor group, and 8 (25%) who had thrombi (Figure 4). The remaining 41 patients (56%) did not receive dipyridamole because they (or their primary physician) refused (n = 14), had poor clinical condition (n = 24), or because of their young age (n = 3). There were no significant alterations of systolic blood pressure and heart rate from baseline to dipyridamole peak infusion. Values of quantitative RTPE were very low in patients with thrombi. At rest, A value was 0.1 dB (0.01-0.51) and at peak 0.21 dB (0.01-0.73) while A × *β* value were 0.15 dB/s^−1^ (0.01-0.47) and 0.09 dB/s^−1^ (0.01-0.32), respectively.

Among the 24 patients with cardiac tumors who underwent dipyridamole study, two-dimensional echocardiography did not differentiate a benign from malign tumor in 12 (50%) of patients. By qualitative analysis of RTPE, absence of perfusion (score 0) was observed in 2 patients with malignant tumor; mild perfusion (score 1) was observed in 6 (70%) patients with benign tumor and 12 (85%) patients with malignant tumor; moderate perfusion (score 2) was observed in 2 patients (20%) with benign tumor, and intense perfusion (score 3) was observed in 1 (10%) patient with benign tumor. By qualitative analysis, we were not able to differentiate benign from malignant tumors. Table 5 shows quantitative parameters at rest and dipyridamole stress in patients with malignant and benign tumors. No significant differences were found between patients with benign and malignant tumors at baseline. At dipyridamole stress, the group with malignant tumors evidenced greater microvascular blood volume (A) than the group with benign tumors. No difference was evidenced between groups with malignant tumors and benign tumors considering A reserve [0.91 (0.62-1.72) and 0.73 (0.5-1.48), respectively; p = NS) and A × *β* reserve [1.18 (0.36-1.75) and 1.1(0.56-1.82), respectively; p = NS]. Parameters of perfusion analyzed in the 24 patients with tumors showed that when comparing only the groups with malignant and benign tumors, the ROC curve of microvascular blood volume (A) at the peak of dipyridamole stress showed an AUC of 0.75. Therefore, A > 3.28 dB at peak dipyridamole stress RTPE was predictive of malignant tumor. This value had 71% sensitivity, 70% specificity, 77% positive predictive value, 64% negative predictive value and 71% accuracy to predict a malignant tumor. A cardiac tumor with A ≥ 3.28 dB on dipyridamole stress RTPE had 5.8-times higher chance of being malignant rather than benign tumor.

**Table 5:** **Median and quartile values of quantitative perfusion variables at baseline and dipyridamole stress, in patients with malignant and benign tumors**

**Variable**	**Malignant tumors (n = 14)**			**Benign tumors (n = 10)**			
	**P25**	**MEDIAN**	**P75**	**P25**	**MEDIAN**	**P75**	**P**
A (dB) rest	1.7	3.11	8.4	1.11	1.88	4.55	†
Axβ (dB/s^−1^) rest	1.11	1.88	4.55	0.49	1.11	4.52	†
A (dB) - stress	2.14	4.18	7.93	1.11	2.69	4.26	§
Axβ (dB/s^−1^) - stress	1.42	2.46	4.59	0.55	1.55	5.5	†

A: Blood volume; Axβ: Regional microvascular blood flow.

† = not significant between malignant tumors and benign tumors; § = p < 0.05 between malignant tumors and benign tumors.

## Discussion

The diagnosis of cardiac masses is challenging, given the specific characteristics of the disease and the peculiar images that they nearly always generate. Thanks to the advances in cardiovascular imaging modalities in the last 50 years, there has been a noticeable improvement in the knowledge about the prevalence and natural history of cardiac masses. Among these methods, echocardiography has been demonstrated extremely useful for evaluating patients with suspected cardiac masses. Nevertheless, the great limitation of this method resides in its inability to distinguish between thrombi and malignant or benign tumors. The fundamental histologic difference between thrombi and tumors is the intense vascularity of the latter, the thrombi being avascularized or with rare canaliculi inside them. Benign tumors have scarce vascularity, while malignant tumors have abundant neovascularization.

RTPE is a modality that uses no ionizing agent, is versatile and cost-effective study for the evaluation of myocardial perfusion, therefore highly desirable for the evaluation of cardiac mass. In the recent years, its diagnostic and prognostic value has been established in patients with suspected coronary artery disease [7,8,19-21]. Quantitative RTPE has the potential advantage to be less dependent from observer experience and allows for determining myocardial flow reserve. This technique, although time-consuming was shown useful for evaluating different clinical conditions with alteration in microvascular blood flow [17,22-25]. The angiogenic response of tumors varies according to the existing circulation [26,27]. Neovascularization may have an increase in diameter or length, thus varying its internal resistance and consequently the local tumor perfusion. Besides that, there are tumors which receive a direct irrigation from coronary arteries, as it was the case in two myxomas and one paraganglioma among our study patients.

Qualitative analysis of perfusion echocardiography is easy, although its reproducibility is problematic as a reliable method for differentiating cardiac masses. Quantitative analysis of RTPE has the potential to enable the differential diagnosis of tumor from thrombi with higher accuracy. In the present study, masses with microvascular volume (A) < 0.64 dB and microvascular blood flow (A × *β*) < 0.30 dB/s^−1^ predicted thrombi with accuracy of 85% and 87%, respectively. In the literature we found only one report with similar results [9], which was limited to quantification of perfusion of only nine tumor cases, since the other seven masses were thrombi. Parameters of blood flow were compared between the mass and adjacent myocardium.

Of note, although there are previous studies demonstrating the value of contrast echocardiography in patients with cardiac masses [8-12,28], this is the first time in the literature that dipyridamole stress RTPE was used in a high number of patients for evaluating cardiac masses and we demonstrated it to be a safe method. Dipyridamole stress significantly contributed for differentiating malignant from benign cardiac tumors. There was an increase in the value of microvascular blood volume (A) in the group with malignant tumors as compared with that of benign tumors. Cardiac tumors presenting a value A > 3.28 dB in dipyridamole stress RTPE had 5.8-times higher chance of being a malignant rather than a benign tumor. It is well known that malignant tumors have intense neovascularization, vessels with thin, tortuous walls with greater variability in diameter. However, until now there was no study in the literature using dipyridamole in patients with cardiac tumors with the purpose of evaluating tumoral perfusion vascular reactivity. Hence, we believe that these data may serve as the initial trigger for future investigation with new quantification techniques as parametric imaging in order to better define tumoral characteristics.

### Study limitations

Although one potential limitation of the present study is the great variability in etiology of tumors, the distribution among groups was a proportionate one (28% benign cardiac masses, 38% thrombi, and 27% malignant tumors). Not all patients underwent dipyridamole infusion because of refusal to undergo stress or clinical contraindication to exam. Therefore, one could argue that this analysis was incomplete. We would like to note that the total number of patients evaluated by dipyridamole stress RTPE was still reasonable (32) considering the specific patient population studied and the criteria for inclusion in the study. One possible limitation is heterogeneity in quantification data because of the use of different contrast agents (PESDA and Definity). However, we have recently published studies of myocardial perfusion quantification showing the diagnostic and prognostic value of RTPE using both contrast agents in different patient population [17,25]. Another limitation is the high intraobserver variability found in our study. This probably expresses the difficulty of analyzing perfusion in cardiac tumors due to their amorphous, heterogeneous structure and calls for the development of a more observer independent tool for its analysis. Finally, in some cases thrombi or tumors could have been diagnosed with very high degree of probability based solely on their features in standard echocardiography. In these cases perfusion contrast echocardiography did not add value over clinical and two-dimensional echocardiography.

## Conclusion

RTPE allowed for demonstration that cardiac tumors have greater microvascular blood volume and regional blood flow in comparison with thrombi. Qualitative analysis is a fast diagnostic approach to diagnose thrombi, however it is still poorly reproducible. Dipyridamole stress quantitative RTPE was useful for differentiating malignant from benign tumors.

## Abbreviations

A: Peak signal intensity plateau
AUC: Area under the curve
β: Signal intensity rate of rise
PESDA: Perfluorocarbon-exposed sonicated dextrose albumin
ROC: Receiver-operator characteristic
RTPE: Real-time perfusion echocardiography
